# Diagnostic accuracy of an interdisciplinary tertiary center evaluation in children referred for suspected congenital anomalies of the kidney and urinary tract on fetal ultrasound - a retrospective outcome analysis

**DOI:** 10.1007/s00467-021-05139-z

**Published:** 2021-06-14

**Authors:** Barbara Schürch, Gwendolin Manegold-Brauer, Heidrun Schönberger, Johanna Büchel, Olav Lapaire, Annkathrin Butenschön, Evelyn A. Huhn, Dorothy Huang, Katrina S. Evers, Alexandra Goischke, Martina Frech-Dörfler, Christoph Rudin

**Affiliations:** 1 University of Basel, Basel, Switzerland; 2 University Women’s Hospital Basel, Basel, Switzerland; 3 Department of Pediatric Nephrology, University Children’s Hospital Basel, Basel, Switzerland; 4 Department of Pediatric Surgery, University Children’s Hospital Basel, Basel, Switzerland

**Keywords:** Hydronephrosis, CAKUT, AUDAKUT, Kidney malformation, Urinary tract anomaly, Fetal ultrasound

## Abstract

**Background:**

Fetal ultrasound organ screening has become a standard of care in most high-income countries. This has resulted in increased detection of congenital abnormalities, which may lead to major uncertainty and anxiety in expectant parents, even though many of them are of minor relevance. In order to optimize prenatal counselling, we introduced an interdisciplinary approach for all pregnant women referred to our center by private obstetricians for a co-assessment of suspected relevant fetal abnormalities of the kidney or urinary tract, involving both experienced prenatal ultrasound specialists and a pediatric nephrologist or urologist.

**Methods:**

In a retrospective analysis, we evaluated reports of intrauterine evaluation and postnatal follow-up in order to assess accuracy of explicit intrauterine diagnoses and outcome of hydronephroses according to their severity in this setting.

**Results:**

A total of 175 fetuses were examined between 2012 and 2019 and followed postnatally at our Pediatric Nephrology or Urology Department. There was a high concordance (85.9%) between explicit intrauterine and final diagnoses. Resolution rate of hydronephrosis was higher in patients with intrauterine low-grade than high-grade hydronephrosis (61.8% versus 11.9%). An etiological diagnosis was found in 62.5%, 52.0%, and 11.1% of patients with intrauterine bilateral high-grade, unilateral high-grade, and unilateral high-grade with contralateral low-grade hydronephrosis, respectively, but in none of the patients with intrauterine low-grade hydronephrosis.

**Conclusions:**

The results of our study demonstrate that, through interdisciplinary teamwork, intrauterine assessment of the fetal kidneys and urinary tract is highly accurate and allows a good discrimination between relevant and transient/physiological hydronephroses.

**Graphical abstract:**

**Figure:**
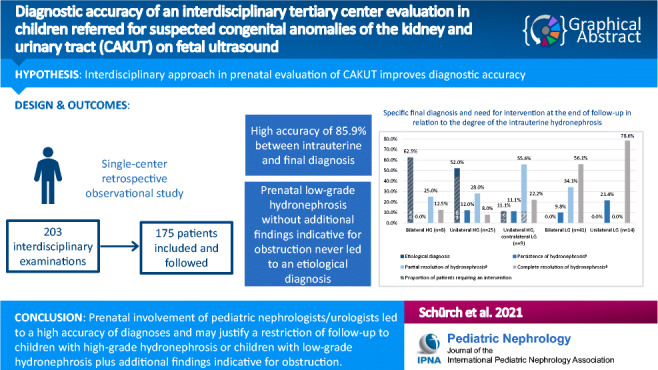
A higher resolution version of the Graphical abstract is available as Supplementary information

**Supplementary Information:**

The online version contains supplementary material available at 10.1007/s00467-021-05139-z.

## Introduction

Fetal ultrasound has become a standard of care in high-income countries and an important instrument for the detection of fetal abnormalities. It allows us to anticipate neonatal needs and to plan delivery and specialized neonatal care in a perinatal center if necessary. Congenital anomalies of the kidney and urinary tract (CAKUT) are detected in 0.2–0.76% of prenatal ultrasound examinations and account for approximately 20% of all fetal congenital defects [1–6].

It is well known that ultrasound imaging is much more operator-dependent than computed tomography or magnetic resonance imaging. This is especially true for obstetric ultrasound imaging, as the small size of fetal organs and the variable fetal position in the uterus create particular challenges [7, 8]. Many studies have addressed the importance of early diagnosis of fetal pathologies due to their impact on outcome after delivery. Several authors stressed the difficulty of collaboration and data exchange between prenatal and postnatal specialists [9–12]. In numerous studies, pediatric nephrologists or urologists seem to be involved only postnatally. Other centers provide prenatal counselling by pediatric nephrologists or urologists only for selected patients [3, 12], whereas yet others leave the decision solely to the expectant parents [13, 14].

In order to optimize the intrauterine assessment of antenatal ultrasound-diagnosed anomalies of the kidney and urinary tract (AUDAKUT), we initiated an interdisciplinary approach at our center in 2012. In case of any prenatal referral to our tertiary perinatal university center or to one single highly specialized private obstetric ultrasound practice for the co-assessment of an urogenital abnormality identified by a private obstetrician, fetal ultrasound was performed by the most experienced prenatal ultrasound specialists in the presence of a pediatric nephrologist or urologist. The aim of this study was to evaluate the impact of this specialized setting by studying the diagnostic accordance between findings on prenatal ultrasound examination and final diagnosis. Furthermore, we assessed whether intrauterine ultrasound findings such as the degree of hydronephrosis can predict the likelihood of detecting etiological pathologies and the need for postnatal interventions or spontaneous resolution over the course of time.

## Methods

### Study design and inclusion criteria

In this single-center retrospective observational study at the University Women’s and Children’s Hospitals of Basel, Switzerland, we describe all children who received a prenatal ultrasound examination due to suspicion of AUDAKUT in this interdisciplinary setting over the period of 7.5 years between January 2012 and June 2019. All expectant mothers were referred to our center for a co-assessment of AUDAKUT suspected by a private obstetrician according to a significantly enlarged antero-posterior kidney pelvis diameter in the transverse plane (APKPD) or a suspected malformation on routine second trimester ultrasound. A total of nine highly experienced prenatal ultrasound specialists performed all ultrasound examinations, seven at the University Women’s Hospital and two in a specialized local private obstetric ultrasound practice. The prenatal ultrasound specialists demonstrated the findings in real time to the head of the Department of Nephrology or Urology of the University Children’s Hospital, who immediately wrote a specific structured protocol after the examination of the findings, likely diagnosis and further management. If rapid postnatal clarification or intervention appeared necessary, delivery was planned at the perinatal center ran by both hospitals in the obstetric unit of the University Women’s Hospital.

**Fig. 1:**
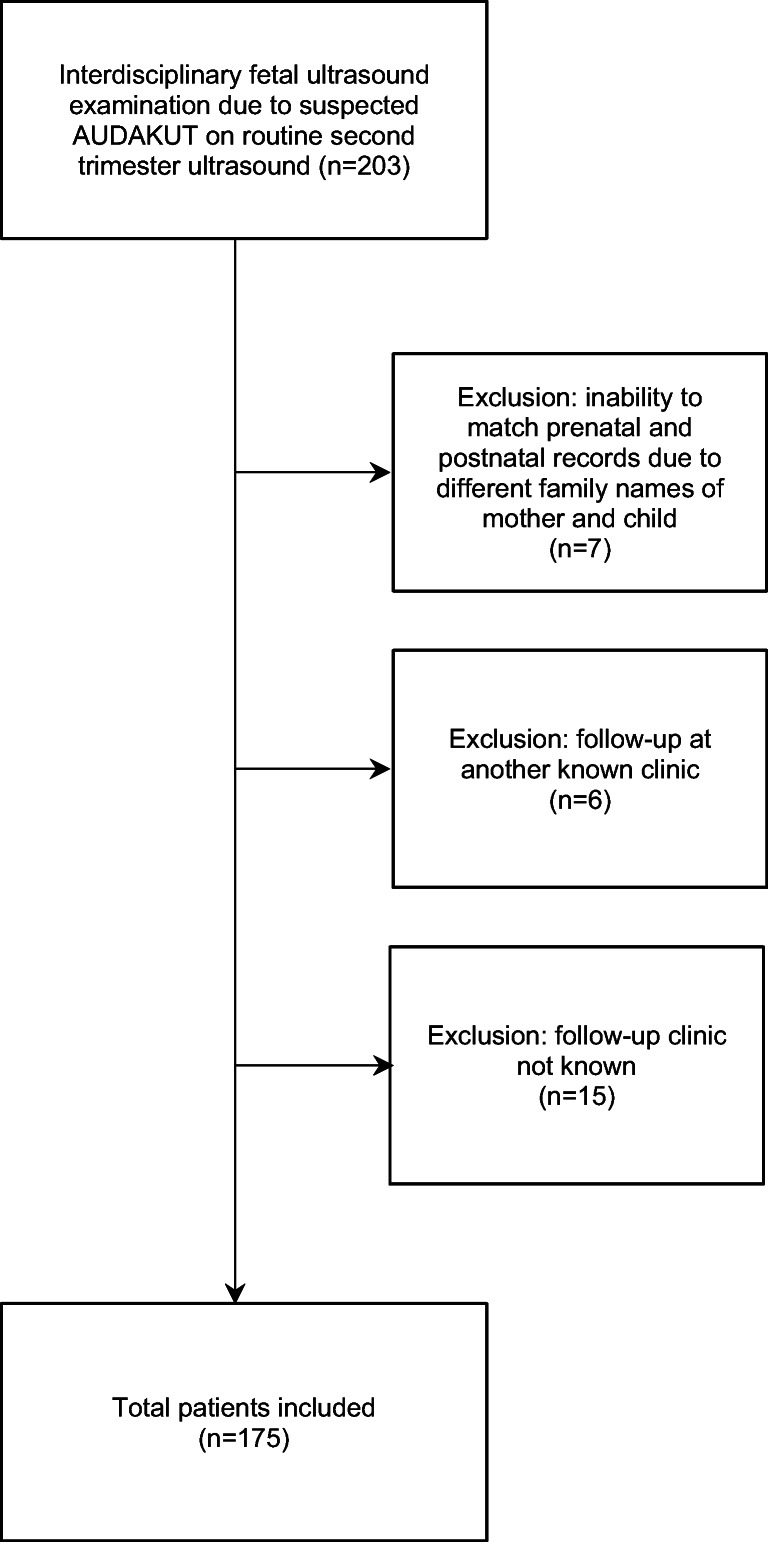
Number of patients included in/excluded from the study and reasons for exclusion

We included all patients for whom such an intrauterine protocol was available and who had been postnatally followed by the Pediatric Nephrology or Urology Units of the University Children’s Hospital. We had to exclude cases with inability to match prenatal and postnatal records (due to different family names of mother and child) and children evaluated and followed elsewhere (see Fig. 1).

### Data collection

Data were extracted from the clinical records of the University Children’s Hospital Basel taken at three different time points: (1) written protocol of the interdisciplinary fetal ultrasound examination, (2) first postnatal radiology report on day 1 and/or 4 and first postnatal clinical report, (3) clinical and radiology reports of the most recent assessment.

In case of more than one prenatal assessment, the diagnosis and point of time of the most recent evaluation were used for analysis. The final diagnosis was defined as the one mentioned in the most recent clinical or radiological report. In patients with normal findings at postnatal investigation and without further evaluations, the final diagnosis was considered as the one mentioned at the postnatal assessment. In patients receiving a first ultrasound examination on the first day of life and a subsequent intervention, the degree of hydronephrosis on day 1 was used for analysis. For all the other patients, the degree of hydronephrosis of the first ultrasound examination from day 3 onward was used, in order to avoid underestimation of the degree of hydronephrosis due to physiological neonatal oliguria.

### Investigations and follow-up

**Fig. 2:**
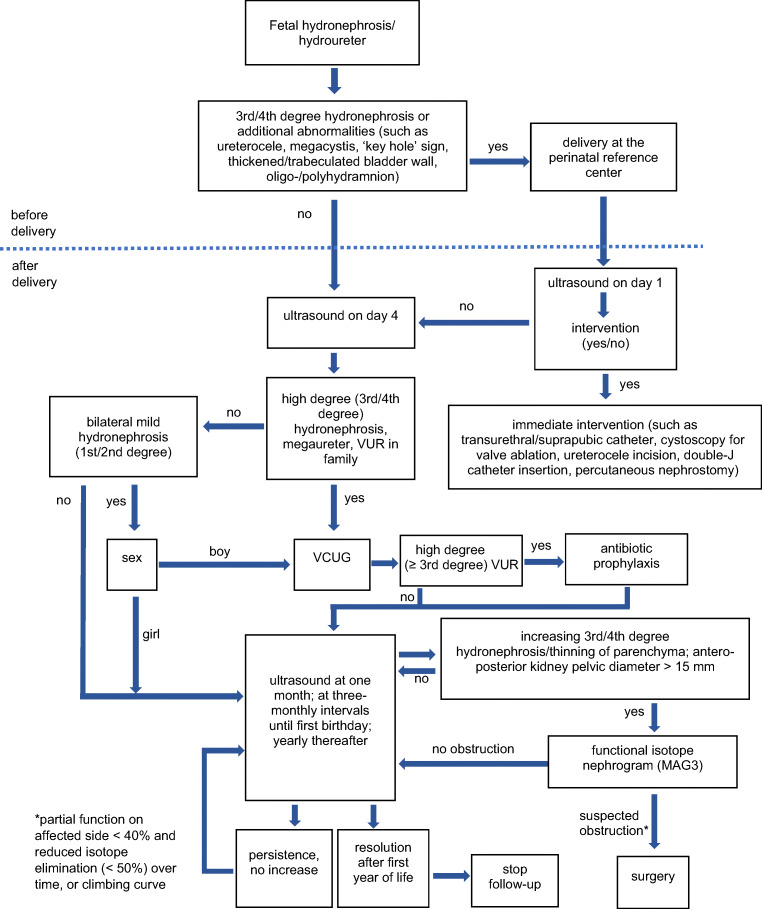
Principles of investigations and follow-up of neonates with fetal/congenital hydronephrosis

There was no specific protocol for investigation and follow-up management, since this was a retrospective analysis and not designed as a clinical trial. However, the children were examined and followed according to the principles summarized in Fig. 2. Data were analyzed in two different ways, namely as a *per patient analysis* (presented herein) and a *per kidney unit analysis* (available in the online Supplementary Information (SI)), using the same definitions for the degree of hydronephrosis and criteria for the outcome.

### Definitions

Hydronephrosis was graded into four degrees according to Beetz et al. [15]. Grade III and IV were considered as high-grade (HG) and grade I and II as low-grade (LG) hydronephrosis. If hydronephrosis was documented as lying between two grades, the higher grade was used for analysis. Partial resolution of hydronephrosis was considered as either (1) a change to a lower subgroup of hydronephrosis, (2) a change from bilateral to unilateral hydronephrosis without change of the subgroup, or (3) a persisting hydroureter in patients with former hydronephrosis.

### Methods

Neonates were divided into two groups according to the result of the prenatal sonographic assessment.

- *Group 1* included all patients with exclusive intrauterine hydronephrosis and/or hydroureter. Patients were further divided into five different subgroups according to the localization (unilateral or bilateral) and the degree of hydronephrosis (HG or LG): (1) bilateral HG, (2) unilateral HG, (3) unilateral HG and contralateral LG, (4) bilateral LG, (5) unilateral LG. In this group, we analyzed whether the intrauterine description or the degree of hydronephrosis was predictive for any final diagnosis on follow-up, such as vesico-ureteral reflux (VUR), posterior urethral valves (PUV), or the presence/development of pelviureteric junction obstruction (PUJO) or vesico-ureteric junction obstruction (VUJO). Furthermore, we analyzed evolution with regard to total or partial resolution, persistence, or progression of hydronephrosis.
- *Group 2* included all patients with an explicit intrauterine diagnosis including kidney agenesis, fusion (horseshoe kidney), ectopy, duplex kidney with and without ureterocele, multicystic dysplastic kidney, or a cystic/dysplastic or hypoplastic kidney. Furthermore, group 2 also included all patients with hydronephrosis plus any additional related pathology of the urinary tract noted on prenatal ultrasound examination, including indirect signs suggestive of PUV in male fetuses, such as megacystis, dilated posterior urethra (so-called keyhole-sign), or a thickened or trabeculated bladder wall. An abnormal amount of amniotic fluid was interpreted as a possible sign for any kind of postrenal obstruction, including PUV, PUJO, or VUJO. In this group, we analyzed the accuracy of an explicit or matching intrauterine diagnosis:

*Concordance was considered as given*:

- If intrauterine and final diagnoses were identical.
- If the prenatal ultrasound examination had shown a hyperechogenic kidney in case of a cystic/dysplastic or hypoplastic kidney.
- If follow-up resulted in a final etiological diagnosis (such as VUR, PUJO, VUJO, PUV, bladder diverticulum, or ureterocele) matching the intrauterine findings in patients with intrauterine hydronephrosis plus an additional related pathology.
- If the intrauterine diagnosis was confirmed on postnatal examinations but an additional anomaly of the kidney or the urinary tract was detected during follow-up; the newly identified pathology except VUR diagnosed during follow-up was listed as an additional diagnosis.
- If patients with suspicion of kidney agenesis due to empty kidney bed on prenatal ultrasound examination showed ectopy during follow-up. Ectopy was listed as an additional diagnosis in these cases.

*Concordance was considered as partial*:

- If one intrauterine diagnosis was confirmed, but a second intrauterine diagnosis was not confirmed during follow-up.
- If the diagnosis was confirmed, but the affected side was not identical in the intrauterine and final diagnoses.

*Concordance was considered as not given*:

- If during follow-up, the intrauterine diagnosis was not confirmed and the final diagnosis was different.

*The intrauterine diagnosis was considered as false positive*:

- If postnatal or follow-up examinations resulted in unremarkable findings.
- If, in patients with hydronephrosis plus an additional related pathology, hydronephrosis persisted or was shown to regress and an etiological diagnosis (VUR, PUJO, VUJO, PUV, bladder diverticulum, or ureterocele) was excluded after investigations by voiding cystourethrography (VCUG) and dynamic isotope nephrogram with mercaptoacetyltriglycine (MAG3) and furosemide.

Positive predictive value was calculated as the percentage of any postnatally confirmed kidney or urinary tract pathology in all children with a respective suspicion on prenatal ultrasound examination.

### Statistics

The extracted data were transferred into a Microsoft Excel sheet. Data were analyzed using descriptive statistics. General information about the study population was described using mean values and standard deviations, and frequencies were described by using percentages.

### Ethics

This study (project ID: 2019-01762) was approved by the Ethics Committee of Northwestern and Central Switzerland (EKNZ).

## Results

### Inclusion and main outcome

A total of 203 interdisciplinary fetal ultrasound examinations for external suspicion of AUDAKUT took place between January 2012 and June 2019, and 175 patients (86.2%) were included in the study (Fig. 1). Apart from one intrauterine fetal death (IUFD) at 34 weeks of gestation due to bilateral kidney agenesis, there was no miscarriage, stillbirth, or induced abortion, nor was there any death postpartum during follow-up. Mean postpartum follow-up period of all patients was 23.2 ± 18.7 (range 0.067–72.9) months. Among 174 live-born children, 38 (21.8%) required an intervention during follow-up, 18 of group 1 and 20 of group 2, respectively. The relative risk (RR) for an intervention in 129 children with intrauterine hydronephrosis of group 1 and 2 was 34.6 in those with HG (33/63) as compared to those with LG hydronephrosis (1/66).

### Patients’ main characteristics

The main characteristics of the children and their mothers are listed in Table 1 and additional information is available in the online Supplementary Information (SI-1 and SI-2).

**Table 1:** Patients’ main characteristics (174 live borns)

			Figures	Number of patients (percentage)		
Sex				*n* = 175		
	Male				105 (60.0%)	
	Female				69 (39.4%)	
	Not known (IUFD)				1 (0.6%)	
	Male-to-female ratio		1.52:1			
Duration of pregnancy (weeks of gestation)				*n* = 174		
	Term (≥ 38)				162 (93.1%)	
	Preterm (< 38)				12 (6.9%)	
		35–37				10
		29–34				1
		< 29				1
Birth weight				*n* = 166		
	Mean birth weight (range)		3379 ± 583 g (1490–4790)			
Maternal characteristics				*n* = 175		
	Maternal age (range)		32.4 ± 5.2 years (20–47)			
	Parity					
		P0				93 (53.1%)
		P1				61 (34.9%)
		≥ P2				21 (12.0%)
Positive family medical history for CAKUT^a^				*n* = 166		
	Overall positive family medical history for CAKUT				50 (30.1%)	
		Identical to the anomaly diagnosed in the study subject				10 (6.0%)
		Different to the anomaly diagnosed in the study subject				40 (24.1%)
Associated extrarenal anomalies^b^				*n* = 156		
	No associated anomaly				118 (75.6%)	
	Single associated anomaly				27 (17.3%)	
	Several associated anomalies				11 (7.1%)	

^a^Details about family medical history for CAKUT, see online Supplementary Information 1

^b^Details about associated extrarenal anomalies, see online Supplementary Information 2

In 50 children (30.1%), the family medical history was positive for kidney anomalies, including 7 children with several kidney and urogenital anomalies in their families. In 10 patients (6.0%), the reported pathology was identical to the anomaly diagnosed in the study subject (SI-1).

A total of 38 children (24.4%) showed additional extrarenal anomalies, including 27 (17.3%) with a single anomaly and 11 (7.1%) with several anomalies or a syndrome, either genetically verified or suspected (see Table 1 and for additional details SI-2).

On average, the prenatal ultrasound examination used for analysis took place at 31 (range 21–40) weeks of gestation. Sixteen children had an additional earlier examination, which took place at 24 (range 19–33) weeks of gestation. One child with bilateral HG hydronephrosis had three prenatal ultrasound examinations.

### Group 1

Of the 175 children included in this study, 97 (55.4%) were assigned to group 1. Intrauterine hydronephrosis was bilaterally HG in 8 (8.2%), unilaterally HG in 25 (25.8%), unilaterally HG and contralaterally LG in 9 (9.3%), bilaterally LG in 41 (42.3%), and unilaterally LG in 14 patients (14.4%).

At the end of follow-up, 39 patients (40.2%) showed complete resolution of the hydronephrosis and an unremarkable ultrasound examination, including 11 (78.6%), 23 (56.1%), 2 (22.2%), 2 (8.0%), and 1 patients (12.5%) with prenatal unilateral LG, bilateral LG, unilateral HG with contralateral LG, unilateral HG, and bilateral HG hydronephroses, respectively. In 19 (48.7%) children, complete resolution was already present on first postpartum evaluation (ultrasound on day 1 and/or 4). Resolution rate was much higher in the subgroups with intrauterine LG (61.8%) than in those with HG hydronephrosis (11.9%).

Nineteen (19.6%) patients had a specific etiologic diagnosis and 39 (40.2%) had persistent hydronephrosis including 11 (11.3%) with an unchanged (10 patients) or even higher (1 patient) and 28 (28.9%) with a lower degree.

Outcome of hydronephrosis as well as type and number of final diagnoses of patients in group 1 are listed in Table 2. A further investigation by VCUG was carried out in 50 patients of group 1, and 5 (10%) were found to have VUR. In 3 patients VUR was associated with another diagnosis, while in 2 it was an isolated final diagnosis. Of the 39 patients with either persisting or partially resolved hydronephrosis, 26 underwent VCUG. VUR and PUV (20 boys) were ruled out in all of them. Obstruction was excluded by a dynamic isotope nephrogram in 5 children as well.

**Table 2:** Outcome of hydronephrosis and final diagnoses in patients of group 1 (*n* = 97)

Final etiological diagnosis	Outcome of hydronephrosis/diagnosis			Number of patients (*n* = 97)			Percentage
No				78			80.4%
	*Outcome of hydronephrosis*						
	Complete resolution of hydronephrosis				39		40.2%
		• without associated pathologies				38	
		• with associated ADPKD				1	
	Persistent hydronephrosis				39		40.2%
		• isolated hydronephrosis				33	
		• isolated hydroureter				1	
		• hydronephrosis and hydroureter				5	
Yes				19			19.6%
	*Final etiological diagnosis*						
	PUJO				9		
		• isolated				7	
		• + VUJO				1	
		• + cystic dysplastic kidney				1	
	Duplex kidney				2		
		• isolated				0	
		• + ureterocele				1	
		• + VUR				1	
	Urethral valves				3		
		• isolated				1	
		• + VUR				1	
		• + VUR + PUJO				1	
	VUR				2		
	Adult ureterocele				1		
	VUJO				1		
	Ureteral stenosis				1		

*ADPKD* autosomal dominant polycystic kidney disease, *VUR* vesico-ureteral reflux, *VUJO* vesico-ureteric junction obstruction, *PUJO* pelviureteric junction obstruction

**Fig. 3:**
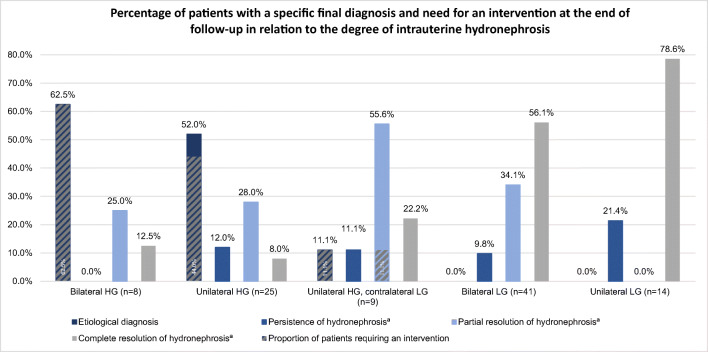
Specific final diagnosis and need for intervention at the end of follow-up in relation to the degree of the intrauterine hydronephrosis (per patient analysis of group 1) (for the per kidney unit analysis, see Supplementary Information 3). ^a^Without etiological diagnosis. *LG hydronephrosis* low-grade hydronephrosis (grades I and II according to the grading system of Beetz et al. [15]), *HG hydronephrosis* high-grade hydronephrosis (grades III and IV according to the grading system of Beetz et al. [15])

Figure 3 summarizes the outcome and rate of interventions in 97 patients of group 1 at the end of follow-up in relation to the degree of their intrauterine hydronephrosis.

A specific etiological diagnosis was made in 19 patients (19.6%). All these children showed intrauterine HG hydronephrosis (5 patients with bilateral HG, 13 with unilateral HG, and 1 with unilateral HG and contralateral LG hydronephrosis). Seventeen of these patients required an intervention, whereas 2 (one with an adult ureterocele and one with VUR) did not. Furthermore, one boy with intrauterine unilateral HG and contralateral LG hydronephrosis received diagnostic cystoscopy after an unsuccessful attempt to insert a transurethral catheter. No infravesical obstruction was found, and in consequence this patient belongs to those with persistent hydronephrosis without final etiological diagnosis. Taking this patient into account, a total of 18 patients in group 1 required an intervention. Intervention was carried out in the first week of life in 6 (33.3%) of these 18 patients due to bilateral (2 patients) or unilateral (4 patients) HG hydronephrosis in association with findings suggestive for urinary tract obstruction on ultrasound examination on the first day of life.

Three male patients with intrauterine bilateral (2 boys) or unilateral (1 boy) HG hydronephrosis without any additional findings or indirect signs indicative for PUV were found to have urethral valves when further investigated by VCUG, retrograde pyelography, or at urethrocystoscopy for other reasons. One of these 3 patients had trisomy 21 (diagnosed during fetal life), a second also had bilateral VUR, and the third showed unilateral PUJO and bilateral VUR during follow-up.

In contrast to children with HG hydronephrosis, no etiological diagnoses were made until the end of follow-up in patients with intrauterine bilateral or unilateral LG hydronephrosis.

For the results of the *per kidney unit analysis*, see online Supplementary Information (SI-3 including Figures 3b^1^ and 3b^2^).

### Group 2

A total of 78 patients (44.6%) were assigned to group 2. The final diagnosis corresponded to the intrauterine diagnosis in 63 patients (80.8%) and there was partial concordance in 4 (5.1%). Ten patients (12.8%) were classified as false positive and 1 (1.3%) had a different pathology than mentioned in the prenatal protocol. This corresponds to a positive predictive value of 87.2%.

Among the 63 patients (80.8%) with agreement of intrauterine diagnosis during follow-up, 51 patients (65.4%) showed concordance without any additional findings during follow-up and 12 (15.4%) received an additional diagnosis. Type and number of pathologies in patients of group 2 are listed in Table 3 (and with additional details in SI-4).

**Table 3:** Final diagnoses and concordance with intrauterine diagnoses in patients of group 2 (*n* = 78) (for details see electronic Supplementary Information 4)

Concordance^a^	Final diagnosis	Number of patients (n=78) and percentage		
Yes/Given		63		80.8%
	ADPKD		1	
	Agenesis		11	
	Ectopy		11	
	Multicystic dysplastic kidney		15	
	Duplex kidney		16	
	PUJO		1	
	Urethral valves		4	
	VUR		2	
	Cystic dysplastic kidney		1	
	Ureterocele		1	
Yes/Partial		4		5.1%
	Ureterocele		1	
	Duplex kidney		2	
	Multicystic dysplastic kidney		1	
No/Different Pathologies		1		1.3%
	VUR		1	
No/False Positives		10		12.8%
	No pathology		7	
	Hydronephrosis		2	
	Hydroureter		1	

*ADPKD* autosomal dominant polycystic kidney disease, *PUJO* pelviureteric junction obstruction, *VUR* vesico-ureteral reflux

^a^For definitions see “Methods” section

Of the 12 patients with additional diagnoses, 5 patients — prenatally suspected as having kidney agenesis due to an empty kidney bed — showed ectopy of the “missing” kidney on postnatal ultrasound examinations. Four patients showed unrecognized duplex kidneys and 2 patients with fetally diagnosed duplex kidneys were recognized to have ureteroceles only after birth. In the last patient of this subgroup, who developed chronic kidney failure on follow-up, cystic dysplastic kidney turned out to be bilateral instead of unilateral. However, cystic dysplastic alterations of the second kidney might only have become visible between the prenatal and postnatal ultrasound examination.

Among the 4 patients with partial concordance, this was due to suspected duplex kidney, which could not be confirmed on postnatal ultrasound examinations (2 patients) or was located on the contralateral side (1 patient). The fourth child showed a convolute of several cysts interpreted as a cystic-dysplastic upper pole of a duplex kidney on fetal ultrasound. The postnatal ultrasound examination confirmed the duplex kidney but also showed an ureterocele causing HG hydronephrosis of the upper pole. Cystic alterations were most likely mimicked by HG hydronephrosis already present on prenatal ultrasound examination.

Among the 10 patients (12.8%) classified as false positive, 5 were suspected of having duplex kidneys on prenatal ultrasound examination and 1 showed an unclear cystic structure in the bladder. Postnatal ultrasound examination showed normal findings of kidneys and urinary tract in these 6 patients. The other 4 patients belonged to those with intrauterine hydronephrosis and additional pathological findings. In 3 of them with intrauterine HG hydronephrosis, hydronephrosis or hydroureter was persistent or improving during follow-up and VCUG showed normal findings in all of them. An isotope nephrogram was conducted in 2, showing unremarkable findings as well. In the fourth patient with intrauterine bilateral LG hydronephrosis and polyhydramnios, the hydronephrosis had completely resolved on postnatal evaluation and during follow-up.

The patient (1.3%) with a different pathology than the one mentioned in the prenatal protocol had a suspicion of a multicystic dysplastic kidney. Postnatal ultrasound examination was normal. The patient was then followed at another clinic but was later referred again to our clinic after an episode of pyelonephritis and was found to have ipsilateral LG VUR on VCUG.

Altogether, 20 live-born patients (26.0%) in group 2 required one (8 patients) or more (12 patients) interventions. In 9 (45.0%) of these 20 patients, interventions took place during the first week of life. These 9 patients belong to a total of 15 patients who received a first postnatal ultrasound examination on the first day of life. The reasons for this early ultrasound examination were additional findings suggestive of the presence of PUV (9 patients), prenatal suspicion of ureterocele (4 patients), and evaluation of cystic structures of the kidneys (2 patients).

## Discussion

CAKUT are detected in 0.2–0.76% of prenatal ultrasound examinations and account for approximately 20% of all fetal congenital defects [1–6]. Introduction of ultrasound screening programs during the last decades has led to increasing numbers of diagnoses of urinary tract anomalies [3, 16, 17]. This increase is mainly attributable to the detection of more patients with mild hydronephrosis [3, 18], which seems to be physiological and transient in most cases [14, 19]. Such findings may create unnecessary anxiety in expectant parents. However, follow-up is necessary, as patients with LG intrauterine hydronephrosis are also at an increased risk for postnatal kidney pathologies as compared to the general population [20]. In a study conducted by Cordero et al., prenatal consultation with a pediatric urologist was a predictor for a successful follow-up, whereas other factors such as proximity to the pediatric urology service or the severity of prenatal hydronephrosis did not improve adherence to postnatal follow-up [12]. All these factors stress the possible importance of prenatal counselling by pediatric nephrologists or urologists. In everyday practice, counselling may be complicated by insufficient communication and data exchange between pre- and postnatally involved specialists [9, 11]. This was the reason to bring both together as early as possible and to choose an interdisciplinary approach to further improve counselling of expectant parents at our center.

Hydronephrosis represents the most common fetal kidney abnormality, accounting for 51.1–78.9% of all prenatal CAKUT in the literature [4, 6, 21]. This corresponds to our study, in which 73.7% (129 of 175 patients) presented with hydronephrosis, 97 of group 1 and 32 in group 2. It is well accepted and documented in the literature that underlying etiologies and outcome of hydronephrosis strongly correlate with the degree of hydronephrosis.

In many cases, mainly LG fetal hydronephrosis has been shown to be transient and to represent a physiological phenomenon [14, 19, 22]. Transient hydronephrosis has been reported in 47.0–72.0% of patients and in 62.0–87.2% of kidney units in the literature [2, 10, 14, 19], strongly dependent upon the degree of hydronephrosis [2, 4, 19, 22, 23]. In our study complete resolution of hydronephrosis occurred in 78.6% of all patients with unilateral LG and 56.1% of all patients with bilateral LG hydronephrosis in group 1. Even resolution during pregnancy [11] and of HG hydronephrosis [23] has been reported in the literature, and both were also observed in our study.

Similar to the correlation between low degree of hydronephrosis and spontaneous resolution, there is an association between a higher degree of intrauterine hydronephrosis and the likelihood of an etiological urologic pathology [10, 20, 23, 24]. In our study, this was the case in 62.5% of all patients with intrauterine bilateral HG, in 52.0% of those with unilateral HG hydronephrosis, and in 11.1% of those with unilateral HG and contralateral LG hydronephrosis in group 1. In contrast, none of the patients with bilateral or unilateral LG hydronephrosis in group 1 received an etiological diagnosis during follow-up. This is in agreement with the systematic review conducted by Hothi et al., where diagnosis of obstruction in patients with mild-to-moderate kidney pelvis dilatation was rare [25].

In most studies, APKPD has been used for the risk assessment of hydronephrosis. Threshold values of at least 10 mm in the third trimester of pregnancy and at least 12 mm at first postnatal ultrasound examination have been recommended for intensive follow-up by Andres-Jensen et al. [2], who conducted the largest study of AUDAKUT in an unselected birth cohort in a Western country. Instead of the APKPD, we used the four categories of dilatation described by Beetz et al. [15] and a structured description of additional aspects of the kidneys and urinary tract in our study. Even though not directly comparable, our results are in line with those of Andres-Jensen et al. [2], and distinction of LG and HG hydronephrosis as used in our study resulted in a very good discrimination between relevant and irrelevant findings.

Given the continuous increase in morbidity with increasing APKPD, the discussion about optimal limits for relevant diameters at different time points during pregnancy and postnatally is still ongoing. The Society for Fetal Urology (SFU) proposed the SFU Grading of Infant Hydronephrosis system [26], which is similar to the criteria we used. This includes aspects such as calyceal dilatation, thinning and structure of the kidney parenchyma, ureters, bladder, amniotic fluid, and fetal sex. In 2014, an interdisciplinary expert panel also proposed the evaluation of 6 additional sonographic features in addition to the APKPD [27].

As an example, a study of Mallik et al. showed that changing criteria for further follow-up from APKPD ≥ 7 mm or other urinary tract abnormalities, like ureteric or calyceal dilatation, to the only criterion of APKPD ≥ 10 mm would have led to missing diagnoses in 25% of PUJO and 50% of VUR [3]. Thus, even though APKPD remains the most established parameter of hydronephrosis, other factors are likely to add value to intrauterine and postpartal assessment.

Children with intrauterine hydronephrosis have an increased risk of VUR compared to the general population [20]. The degree of hydronephrosis seems not to correlate with the risk of VUR [10, 20]. Routine VCUG in patients with intrauterine hydronephrosis showed VUR in 61% of all patients with normal postnatal ultrasound scan in a study by Jaswon [13]. There was no routine VCUG in our study and we became more restrictive about the indication in recent years. Nevertheless, 50 of the 97 patients of group 1 received a VCUG during follow-up, but only 5 turned out to have VUR. Given its invasive nature, radiation exposure, and the lack of clinical relevance of low-grade VUR, we recommend a rather restrictive use of VCUG as provided by our diagnostic algorithm (Fig. 2) — a strategy supported by others [3, 25]. This is also supported by Ismaili et al., who showed spontaneous resolution of VUR after 1 year in 70% of patients with mild-to-moderate hydronephrosis [28]. Furthermore, patients with intrauterine hydronephrosis and VUR seem to have a better resolution rate of VUR compared with patients first discovered to have VUR after a febrile urinary tract infection [20]. Nevertheless, all parents of these children should be made aware of the signs and symptoms of urinary tract infection, such as fever, irritability, poor feeding, or failure to thrive.

Considering our results, a restriction of follow-up to children with HG hydronephrosis or LG hydronephrosis with additional pathologies (in particular indicative for an obstructive uropathy) seems justifiable. If this approach had been applied in our study, no pathology would have been missed, and 55 patients (56.7%) of group 1 would not have needed any follow-up. This is in line with the recommendations of Andres-Jensen et al. [2] who suggest restricting intensive follow-up to patients who surpass their recommended thres-hold values of APKPD. However, more studies are needed to validate such follow-up restrictions for use in clinical practice.

In our setting, intrauterine diagnosis was confirmed during follow-up in 85.9% of patients in group 2, whereas it was false positive or wrong in only 14.1%. Comparison with the available literature is difficult due to different approaches and interpretations of results. One comparable study conducted by Policiano et al. showed concordance of intrauterine with postnatal diagnoses in 88.8% of cases [6]. However, evaluation in their study also included patients with transient hydronephrosis. Using the same approach in our study would have led to an even higher concordance between intrauterine and final diagnoses.

Some fetal diagnoses seem to be especially challenging. In the literature, ectopic kidneys considerably contribute to the number of missed diagnoses [4]. Prenatal detection rates of only 56% are reported [17]. In our study ectopic kidneys were often missed and mistaken for kidney agenesis (5 out of 11 children), an aspect reported before [6]. This is not surprising given the fact that the fetal position varies and target anatomic regions may be obscured during fetal ultrasound. Duplex kidneys are another diagnosis often missed or misdiagnosed in our study. Prenatal detection rates of only 20.5% have been reported in the literature [22]. Ureteroceles as well were not always diagnosed in our study. We suggest that a large ureterocele might completely fill out an empty bladder and therefore be missed on fetal ultrasound. Finally, the differentiation of cystic structures (including multicystic dysplastic kidney) and hydronephrosis and vice versa can be challenging in some cases, which has also been described in the literature [29, 30]. Of note, some authors described children with unremarkable findings being mistaken for cystic dysplastic kidneys [11]. This is clearly problematic, as expectant parents may be counseled inappropriately about the poor prognosis of this disease in a child who is in fact healthy.

We calculated a positive predictive value for a correct intrauterine suspicion of a kidney or urinary tract pathology of 87.2% in our study. This was higher than the results of other groups, which ranged from 81.5 to 85% [31, 32].

The sensitivity of intrauterine diagnosis for kidney and urinary tract anomalies ranges between 35.7 and 88.5% in the literature [17, 32–34], but could not be calculated in our study due to the unknown number of false-negative cases.

The results of our study demonstrate a high concordance between the intrauterine and final diagnoses in this specialized setting. Missing concordance and false-positive results were mainly due to difficulty in establishing a correct diagnosis with duplex kidneys and in differentiation between cystic alterations and hydronephrosis. Of note, missing concordance never occurred to the disadvantage of patients with serious pathologies.

The overall intervention rate in our study was 21.8%, which is in agreement with the literature, where intervention rates range from 7 and 28% [2, 3, 18, 34]. The proportion of children who needed an intervention immediately after birth or during follow-up in our study was highest in those with the most severe forms of hydronephrosis, which is in agreement with the literature [2, 6, 19]. Our results show a relative risk for an intervention of 34.6 in patients with HG hydronephrosis in comparison to patients with LG hydronephrosis on intrauterine ultrasound examination. Nef et al. showed that one third of the patients included in their study showed normalization of ultrasound findings, one third underwent surgery, and one third had persistent anomalies not requiring surgery [9]. This is similar to the results of our study and underlines the generally good prognosis of most patients with suspicious kidney or urinary tract findings on fetal ultrasound examination.

In correlation with an increased intrauterine detection rate of AUDAKUT, there might be a rising number of terminations of pregnancy [35]. This is supported by the fact that in countries with no routine ultrasound screening policies, the prenatal detection rate of congenital anomalies and number of terminations of pregnancy are lowest [17]. It has been shown that false-positive fetal diagnoses may lead to termination of pregnancy in children with unremarkable findings at autopsy [31, 33, 35]. This might reveal one disadvantage of prenatal screening programs. In contrast to other reports [17, 30], there were no terminations of pregnancy and prevalence of most severe malformations such as bilateral kidney agenesis was low in our study, most probably due to the late referral of the patients to our center. Therefore, earlier terminations of pregnancy cannot be excluded. Nevertheless, we strongly believe that the involvement of pediatric nephrologists or urologists during pregnancy can help to prevent unnecessary terminations of pregnancy.

There are some limitations of this study. Due to its retrospective character, there was no specific study protocol. Twenty-eight patients had to be excluded due to different family names of the mother and the child or follow-up in different hospitals. As we investigated only patients referred to us, we do not know the number of false-negative and true-negative cases and therefore were unable to calculate sensitivity and specificity of our procedure. Unfortunately, APKPD was not systematically measured and documented in our records. APKPD needs to be part of future assessments in order to improve comparability with the literature. Furthermore, it was not possible to deliver accurate data about the frequency of urinary tract infection as this was not systematically recorded during follow-up due to the participation of general pediatric practitioners in patient care.

The main strength of this study is the close collaboration with physical presence of a pediatric nephrologist or urologist at the time of the prenatal examination, which enabled a real-time interdisciplinary assessment and discussion of the ultrasound findings demonstrated by the prenatal ultrasound specialist.

In conclusion, we recommend the prenatal involvement of pediatric nephrologists and urologists for patients with suspicion of AUDAKUT. Our results show that with this approach, there is a high accordance between the intrauterine and final diagnoses, without missing any serious pathology. There was a strong correlation of HG hydronephrosis with causative anatomic pathologies on postnatal investigation and during follow-up. Applying such a specialized setting during pregnancy might justify limiting follow-up examinations to children with HG hydronephrosis or LG hydronephrosis plus additional findings suggestive of urinary tract obstruction. It may also improve expertise in counselling expectant parents and thus lower the risk of loss to follow-up and unnecessary terminations of pregnancy. Reassurance of the expectant parents through prenatal counselling is of key importance, as the majority of fetuses with intrauterine hydronephrosis have a good prognosis and do not require interventions.

Nevertheless, further prospective studies are needed (1) to confirm an additional benefit of prenatal counselling by pediatric nephrologists and urologists, (2) to evaluate feasibility of such a setting within the framework of routine examinations, (3) to calculate sensitivity and specificity of this approach, (4) to further define the optimal intrauterine criteria for the prediction of outcome, and (5) to confirm the possibility to restrict follow-up to certain patients according to intrauterine findings.

## Supplementary information

ESM 1 (DOCX 13 kb).

ESM 2 (DOCX 25 kb).

ESM 3 (DOCX 44 kb).

ESM 4 (DOCX 29 kb).

ESM 5 (PPTX 59 kb).

## Data Availability

The datasets generated and analyzed during the current study are available from the corresponding author on request.

## Abbreviations

ADPKD: autosomal dominant polycystic kidney disease
APKPD: antero-posterior kidney pelvis diameter in the transverse plane
ARPKD: autosomal recessive polycystic kidney disease
AUDAKUT: antenatal ultrasound diagnosed anomalies of the kidney and urinary tract
CAKUT: congenital anomalies of the kidney and urinary tract
EKNZ: Ethics Committee of Northwestern and Central Switzerland
HG: high-grade
IUFD: intrauterine fetal death
LG: low-grade
MAG3: mercaptoacetyltriglycine
PUJO: pelviureteric junction obstruction
PUV: posterior urethral valves
RR: relative risk
SI: Online Supplementary Information
SFU: Society for Fetal Urology
VCUG: voiding cystourethrography
VUJO: vesico-ureteric junction obstruction
VUR: vesico-ureteral reflux
