# Supplementary material for: Diagnostic accuracy of an interdisciplinary tertiary center evaluation in children referred for suspected congenital anomalies of the kidney and urinary tract on fetal ultrasound - a retrospective outcome analysis
Source: Pediatr Nephrol. 2021 Jun 14;36(12):3885–97. doi: 10.1007/s00467-021-05139-z (PMC8599352; doi:10.1007/s00467-021-05139-z)
Supplement: Supplementary file 1 — (DOCX 13 kb). [file 467_2021_5139_MOESM1_ESM.docx]

**Supplementary Information 1**

Article

**Diagnostic accuracy of an interdisciplinary tertiary center evaluation in children referred for suspected congenital anomalies of the kidney and urinary tract on fetal ultrasound – a retrospective outcome analysis**

Barbara Schürch^1^, Gwendolin Manegold-Brauer^2^, Heidrun Schönberger^2^, Johanna Büchel^2^, Olav Lapaire^2^, Annkathrin Butenschön^2^, Evelyn A. Huhn^2^, Dorothy Huang^2^, Katrina S. Evers^3^, Alexandra Goischke^3^, Martina Frech-Dörfler^4^, Christoph Rudin^3^

**Affiliation**

^1^ University of Basel, Basel, Switzerland

^2^ University Women’s Hospital Basel, Basel, Switzerland

^3^ Department of Pediatric Nephrology, University Children's Hospital Basel, Basel, Switzerland

^4^ Department of Pediatric Surgery, University Children's Hospital Basel, Basel, Switzerland

**E-mail address of the corresponding author:**

christoph.rudin@unibas.ch

ORCID: 0000-0002-3789-5915

**Details of positive family medical history of CAKUT (50/166 children) of table 1**

The most frequent pathologies reported were duplex kidney (ten families), obstructive uropathy (six families), kidney agenesis (six families), ectopic kidney (six families), cystic alteration of the kidneys (five families), VUR (four families), autosomal dominant polycystic kidney disease (ADPKD; two families), autosomal recessive polycystic kidney disease (ARPKD; one family), an ureterocele with VUR (one family) and a Potter sequence (one family). Furthermore, three children had a positive family history for a syndrome (Ivemark, Williams and Goldenhar syndrome) and in 12 children it was not possible to determine the exact pathology. In 10 patients (6.0%), the reported pathology was identical to the anomaly diagnosed in the study subject. These anomalies included duplex kidney (four children), multicystic dysplastic kidney (three children), ADPKD (two children) and PUJO (one child).
