# Supplementary material for: Diagnostic accuracy of an interdisciplinary tertiary center evaluation in children referred for suspected congenital anomalies of the kidney and urinary tract on fetal ultrasound - a retrospective outcome analysis
Source: Pediatr Nephrol. 2021 Jun 14;36(12):3885–97. doi: 10.1007/s00467-021-05139-z (PMC8599352; doi:10.1007/s00467-021-05139-z)
Supplement: Supplementary file 2 — (DOCX 25 kb). [file 467_2021_5139_MOESM2_ESM.docx]

**Supplementary Information 2**

Article

**Diagnostic accuracy of an interdisciplinary tertiary center evaluation in children referred for suspected congenital anomalies of the kidney and urinary tract on fetal ultrasound – a retrospective outcome analysis**

Barbara Schürch^1^, Gwendolin Manegold-Brauer^2^, Heidrun Schönberger^2^, Johanna Büchel^2^, Olav Lapaire^2^, Annkathrin Butenschön^2^, Evelyn A. Huhn^2^, Dorothy Huang^2^, Katrina S. Evers^3^, Alexandra Goischke^3^, Martina Frech-Dörfler^4^, Christoph Rudin^3^

**Affiliation**

^1^ University of Basel, Basel, Switzerland

^2^ University Women’s Hospital Basel, Basel, Switzerland

^3^ Department of Pediatric Nephrology, University Children's Hospital Basel, Basel, Switzerland

^4^ Department of Pediatric Surgery, University Children's Hospital Basel, Basel, Switzerland

**E-mail address of the corresponding author:**

christoph.rudin@unibas.ch

ORCID: 0000-0002-3789-5915

**Detail of associated extrarenal anomalies (n=156) of table 1**

Patients' main characteristics (174 live borns)

| **ASSOCIATED ANOMALIES** | |  |  |  | **Number of patients (percentage)** | | | | |
| --- | --- | --- | --- | --- | --- | --- | --- | --- | --- |
|  |  | **Anomaly** | **Detail** |  |  |  |  |  |  |
| **NO** |  |  |  |  | **118** | **75.6%** |  |  |  |
| **YES** |  |  |  |  | **38** | **24.4%** |  |  |  |
|  | **Single anomaly^a^** |  |  |  | 27 | 17.3% |  |  |  |
|  |  | Cardiac anomalies |  |  |  |  | 11 [14] |  |  |
|  |  | Hemangioma |  |  |  |  | 5 [6] |  |  |
|  |  | Genital anomalies |  |  |  |  | 4 [7] |  |  |
|  |  |  | Hypospadia |  |  |  |  | 1 [2] |  |
|  |  |  | Cryptorchidism |  |  |  |  | 2 [5] |  |
|  |  |  | Suspected uterus didelphys |  |  |  |  | 1 |  |
|  |  | Caudal regression sequence |  |  |  |  | 1 |  |  |
|  |  | Sacral dimple |  |  |  |  | 1 |  |  |
|  |  | Pectus excavatum |  |  |  |  | 1[2] |  |  |
|  |  | Clubfoot |  |  |  |  | 1 |  |  |
|  |  | Neonatal tooth |  |  |  |  | 1 |  |  |
|  |  | Supernumerary nipple |  |  |  |  | 1 |  |  |
|  |  | Hip dysplasia |  |  |  |  | 1 |  |  |
|  | **Several anomalies** |  |  |  | 11 | 7.1% |  |  |  |
|  |  | Confirmed syndrome by genetic testing |  |  |  |  | 3 |  |  |
|  |  |  | 22q11.21 deletion syndrome |  |  |  |  | 1 |  |
|  |  |  | Trisomy 21 |  |  |  |  | 1 |  |
|  |  |  | Adams-Oliver syndrome |  |  |  |  | 1 |  |
|  |  | Suspected syndrome |  |  |  |  | 6 |  |  |
|  |  |  | Beckwith-Wiedemann syndrome |  |  |  |  | 1 |  |
|  |  |  | VACTERL association |  |  |  |  | 1 |  |
|  |  |  | MURCS association |  |  |  |  | 1 |  |
|  |  |  | PELVIS syndrome |  |  |  |  | 1 |  |
|  |  |  | Suspected syndrome in patients with facial dysmorphia | |  |  |  | 2 |  |
|  |  |  |  | Isolated facial dysmorphia |  |  |  |  | 1 |
|  |  |  |  | Additional ventricular septal defect |  |  |  |  | 1 |
|  |  | Pectus excavatum and cryptorchidism |  |  |  |  | 1 |  |  |
|  |  | Hypospadia and cryptorchidism |  |  |  |  | 1 |  |  |

^a^ Numbers in squared brackets [] include the respective associated anomalies observed in children with several associated anomalies (patients with suspected or confirmed syndromes and more than one associated anomaly)
