# Supplementary material for: Diagnostic accuracy of an interdisciplinary tertiary center evaluation in children referred for suspected congenital anomalies of the kidney and urinary tract on fetal ultrasound - a retrospective outcome analysis
Source: Pediatr Nephrol. 2021 Jun 14;36(12):3885–97. doi: 10.1007/s00467-021-05139-z (PMC8599352; doi:10.1007/s00467-021-05139-z)
Supplement: Supplementary file 3 — (DOCX 44 kb). [file 467_2021_5139_MOESM3_ESM.docx]

**Supplementary Information 3**

Article

**Diagnostic accuracy of an interdisciplinary tertiary center evaluation in children referred for suspected congenital anomalies of the kidney and urinary tract on fetal ultrasound – a retrospective outcome analysis**

Barbara Schürch^1^, Gwendolin Manegold-Brauer^2^, Heidrun Schönberger^2^, Johanna Büchel^2^, Olav Lapaire^2^, Annkathrin Butenschön^2^, Evelyn A. Huhn^2^, Dorothy Huang^2^, Katrina S. Evers^3^, Alexandra Goischke^3^, Martina Frech-Dörfler^4^, Christoph Rudin^3^

**Affiliation**

^1^ University of Basel, Basel, Switzerland

^2^ University Women’s Hospital Basel, Basel, Switzerland

^3^ Department of Pediatric Nephrology, University Children's Hospital Basel, Basel, Switzerland

^4^ Department of Pediatric Surgery, University Children's Hospital Basel, Basel, Switzerland

**E-mail address of the corresponding author:**

christoph.rudin@unibas.ch

ORCID: 0000-0002-3789-5915

**Per kidney unit analysis**

***Methods***

For the *per kidney unit analysis*, we included every single kidney with intrauterine hydronephrosis of all patients of group 1 and 2, and analyzed whether the degree of hydronephrosis was predictive for the outcome regarding etiological diagnoses (such as VUR, PUJO, VUJO, PUV, bladder diverticulum or ureterocele) as well as resolution or persistence of hydronephrosis.

***Results: Analysis of kidney units of group 1 and 2***

Taking into account all kidney units with intrauterine hydronephrosis of group 1 and 2, an etiological cause was identified in 45 of 78 (57.7%) kidney units with fetal HG hydronephrosis, whereas this was only the case in four out of 123 (3.3%) of those with fetal LG hydronephrosis (figure 3b^1^). Two of these latter children had a HG hydronephrosis of the contralateral kidney and received the diagnoses of PUV with contralateral duplex kidney and of bilateral PUJO with contralateral VUJO and bilateral cystic-dysplastic kidney, respectively. The third patient showed a duplex kidney with ureterocele on follow-up and the last one was found to have unilateral low-grade VUR. All of these four patients belonged to group 2 due to additional findings. Hydronephrosis completely resolved in 88 of 123 (71.5%) kidney units with intrauterine LG and 11 of 78 (14.1%) kidney units with intrauterine HG hydronephrosis.

When we restricted this analysis to kidney units of group 1 (patients with isolated hydronephrosis without additional findings) LG hydronephrosis never led to a final diagnosis or an intervention (figure 3b^2^).

**Fig. 3 (Supplement)** Specific final diagnosis and need for intervention at the end of follow-up in relation to the degree of the intrauterine hydronephrosis (per kidney unit analysis)

^a^ without etiological diagnosis

LG hydronephrosis = low-grade hydronephrosis (grade I and II according to the grading system of Beetz et al. [15]);

HG hydronephrosis = high-grade hydronephrosis (grade III and IV according to the grading system of Beetz et al. [15])
