# Supplementary material for: Diagnostic accuracy of an interdisciplinary tertiary center evaluation in children referred for suspected congenital anomalies of the kidney and urinary tract on fetal ultrasound - a retrospective outcome analysis
Source: Pediatr Nephrol. 2021 Jun 14;36(12):3885–97. doi: 10.1007/s00467-021-05139-z (PMC8599352; doi:10.1007/s00467-021-05139-z)
Supplement: Supplementary file 4 — (DOCX 29 kb). [file 467_2021_5139_MOESM4_ESM.docx]

**Supplementary Information 4**

Article

**Diagnostic accuracy of an interdisciplinary tertiary center evaluation in children referred for suspected congenital anomalies of the kidney and urinary tract on fetal ultrasound – a retrospective outcome analysis**

Barbara Schürch^1^, Gwendolin Manegold-Brauer^2^, Heidrun Schönberger^2^, Johanna Büchel^2^, Olav Lapaire^2^, Annkathrin Butenschön^2^, Evelyn A. Huhn^2^, Dorothy Huang^2^, Katrina S. Evers^3^, Alexandra Goischke^3^, Martina Frech-Dörfler^4^, Christoph Rudin^3^

**Affiliation**

^1^ University of Basel, Basel, Switzerland

^2^ University Women’s Hospital Basel, Basel, Switzerland

^3^ Department of Pediatric Nephrology, University Children's Hospital Basel, Basel, Switzerland

^4^ Department of Pediatric Surgery, University Children's Hospital Basel, Basel, Switzerland

**E-mail address of the corresponding author:**

christoph.rudin@unibas.ch

ORCID: 0000-0002-3789-5915

**Detail of table 3 of the manuscript**

**Table 3** Final diagnoses and concordance with intrauterine diagnoses in patients of group 2 (n=78)

| **CONCORDANCE^#^** | **Final diagnosis** | **Additional details/ findings** | **Suspected intrauterine diagnosis^&^** | **Additional diagnoses** | **Number of patients**  **(n=78) and percentage** | | | |
| --- | --- | --- | --- | --- | --- | --- | --- | --- |
| **YES/GIVEN** |  | | | | **63** |  |  | **80.8%** |
|  | ADPKD | | | |  | 1 |  |  |
|  | Agenesis | | | |  | 11 |  |  |
|  |  | ul | 🗸 |  |  |  | 9 |  |
|  |  | bl | 🗸 |  |  |  | 1 |  |
|  |  | + cl DK | 🗸 |  |  |  | 1 |  |
|  | Ectopy | | | |  | 11 |  |  |
|  |  | isolated | 🗸 |  |  |  | 6 |  |
|  |  |  | agenesis | + il ectopy^a^ |  |  | 3 |  |
|  |  |  | agenesis | + il ectopy and cl DK^a^ |  |  | 1 |  |
|  |  |  | agenesis | + il ectopy and md kidney^a^ |  |  | 1 |  |
|  | Multicystic dysplastic kidney | | | |  | 15 |  |  |
|  |  | isolated | 🗸 |  |  |  | 12 |  |
|  |  | + VUR | 🗸 |  |  |  | 1 |  |
|  |  | + bladder diverticulum | 🗸 |  |  |  | 1 |  |
|  |  |  | 🗸 | + cl DK |  |  | 1 |  |
|  | Duplex kidney | | | |  | 16 |  |  |
|  |  | isolated | 🗸 |  |  |  | 7 |  |
|  |  | + ureterocele | 🗸 |  |  |  | 4 |  |
|  |  | + ureterocele + VUR | 🗸 |  |  |  | 3 |  |
|  |  | + cystic dysplastic upper pole | DK with cystic dysplastic upper pole | + ureterocele |  |  | 1 |  |
|  |  |  | 🗸 | + ureterocele |  |  | 1 |  |
|  | PUJO | | | |  | 1 |  |  |
|  |  | + cystic dysplastic kidneys + VUJO | 🗸 |  |  |  | 1 |  |
|  | Urethral valves | | | |  | 4 |  |  |
|  |  | + VUR | 🗸 |  |  |  | 1 |  |
|  |  | + VUR | bl HN with bl hydroureter, megacystis | + DK |  |  | 1 |  |
|  |  | + DK + ureterocele  + VUR | 🗸 |  |  |  | 1 |  |
|  |  |  | bl HN with ul hydroureter, polyhydramnion | + DK |  |  | 1 |  |
|  | VUR | | | |  | 2 |  |  |
|  |  | isolated | 🗸 |  |  |  | 2 |  |
|  | Cystic dysplastic kidney | | | |  | 1 |  |  |
|  |  | bl | ul cystic dysplastic kidney | + cl cystic dysplastic kidney |  |  | 1 |  |
|  | Ureterocele | | | |  | 1 |  |  |
|  |  |  | HN with ureterocele | + DK |  |  | 1 |  |
| **YES/PARTIAL** |  | | | | **4** |  |  | **5.1%** |
|  | Ureterocele | | | |  | 1 |  |  |
|  |  |  | ureterocele with ul DK | + bladder  diverticulum |  |  | 1 |  |
|  | Duplex kidney | | | |  | 2 |  |  |
|  |  | ul | bl DK |  |  |  | 1 |  |
|  |  |  | DK with cystic dysplastic upper pole | + ureterocele |  |  | 1 |  |
|  | Multicystic dysplastic kidney | | | |  | 1 |  |  |
|  |  |  | il md kidney and DK | + cl DK |  |  | 1 |  |
| **NO/DIFFERENT**  **PATHOLOGIES** |  | | | | **1** |  |  | **1.3%** |
|  | VUR | | | |  | 1 |  |  |
|  |  | isolated | md kidney |  |  |  | 1 |  |
| **NO/FALSE**  **POSITIVES** |  | | | | **10** |  |  | **12.8%** |
|  | No pathology | | | |  | 7 |  |  |
|  |  |  | bl HN with polyhydramnion |  |  |  | 1 |  |
|  |  |  | DK |  |  |  | 5 |  |
|  |  |  | unclear cystic structure in bladder |  |  |  | 1 |  |
|  | Hydronephrosis | | | |  | 2 |  |  |
|  |  | Isolated^b^ | ul HN with polyhydramnion |  |  |  | 1 |  |
|  |  | + hydroureter^b^ | bl HN with bl hydroureter, megacystis, thickened bladder wall |  |  |  | 1 |  |
|  | Hydroureter | | | |  | 1 |  |  |
|  |  | Isolated^b^ | bl HN, bl hydroureter and  polyhydramnion |  |  |  | 1 |  |

^#^for definitions see methods; ^&^🗸 = full concordance with final diagnosis

^a^ Patients with suspicion of kidney agenesis due to empty kidney bed on intrauterine
 ultrasound examination showing ectopy during follow-up were considered as concordance
 of diagnosis (empty kidney bed) with the additional diagnosis of ectopy.

^b^ In all these three patients VUR and urethral valves have been excluded.

ul = unilateral; bl = bilateral; cl = contralateral; DK = duplex kidney; il = ipsilateral;
md = multicystic dysplastic; HN = hydronephrosis; VUR = vesicoureteral reflux; PUJO = pelviureteric junction obstruction
