# Supplementary material for: Diagnostic accuracy of an interdisciplinary tertiary center evaluation in children referred for suspected congenital anomalies of the kidney and urinary tract on fetal ultrasound - a retrospective outcome analysis
Source: Pediatr Nephrol. 2021 Jun 14;36(12):3885–97. doi: 10.1007/s00467-021-05139-z (PMC8599352; doi:10.1007/s00467-021-05139-z)
Supplement: Supplementary file 5 — (PPTX 59 kb). [file 467_2021_5139_MOESM5_ESM.pptx]

## Slide 1
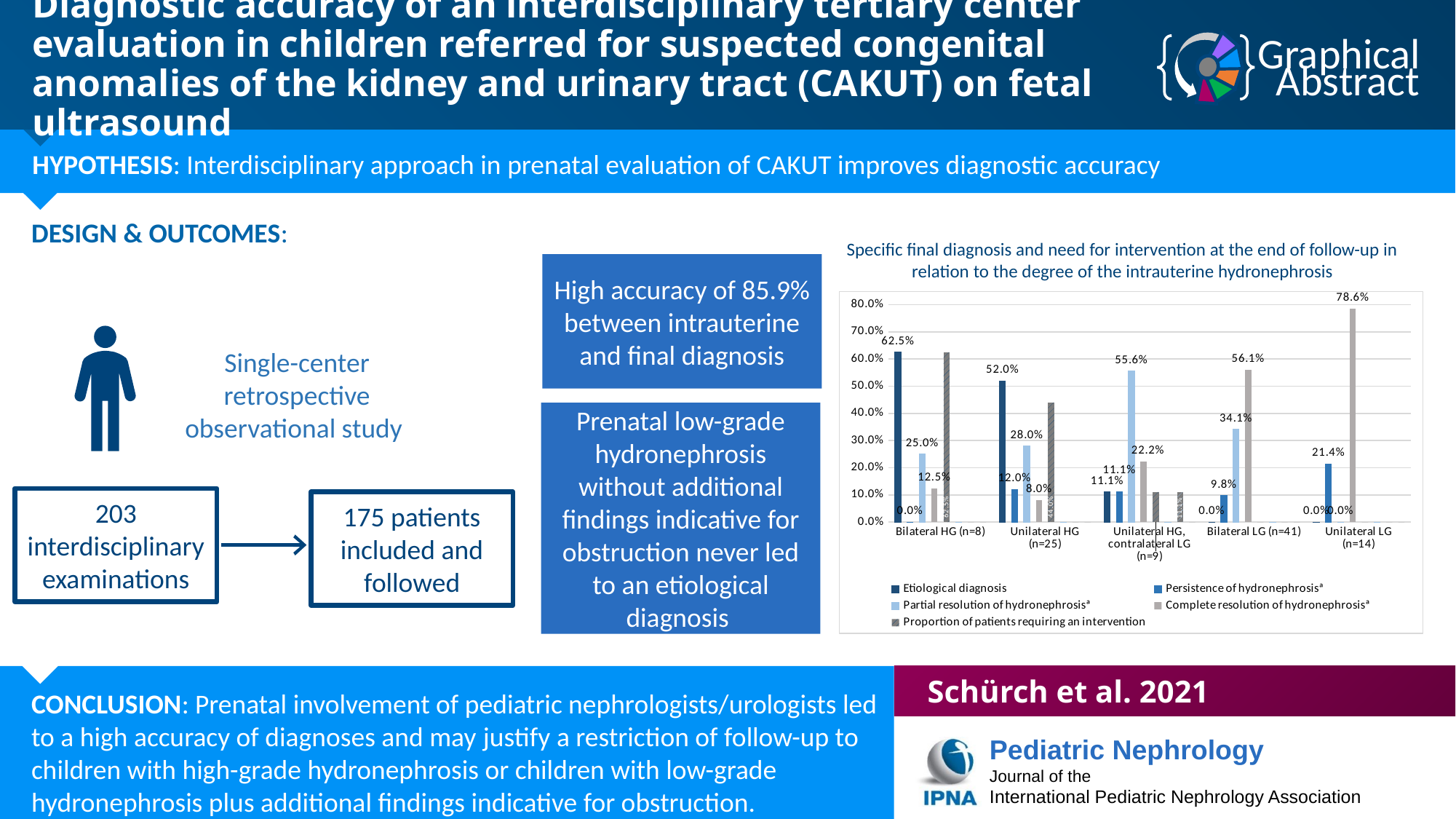

Diagnostic accuracy of an interdisciplinary tertiary center evaluation in children referred for suspected congenital anomalies of the kidney and urinary tract (CAKUT) on fetal ultrasound
HYPOTHESIS: Interdisciplinary approach in prenatal evaluation of CAKUT improves diagnostic accuracy
DESIGN & OUTCOMES:
Specific final diagnosis and need for intervention at the end of follow-up in relation to the degree of the intrauterine hydronephrosis
High accuracy of 85.9% between intrauterine and final diagnosis
### Chart
| Category | Etiological diagnosis | Persistence of hydronephrosisª | Partial resolution of hydronephrosisª | Complete resolution of hydronephrosisª | Proportion of patients requiring an intervention | 022 | 0222 | 03 |
|---|---|---|---|---|---|---|---|---|
| Bilateral HG (n=8) | 0.625 | 0.0 | 0.25 | 0.125 | 0.625 | 0.0 | 0.0 | 0.0 |
| Unilateral HG (n=25) | 0.52 | 0.12 | 0.28 | 0.08 | 0.44 | 0.0 | 0.0 | 0.0 |
| Unilateral HG, contralateral LG (n=9) | 0.111 | 0.111 | 0.556 | 0.222 | 0.111 | 0.0 | 0.111 | 0.0 |
| Bilateral LG (n=41) | 0.0 | 0.098 | 0.341 | 0.561 | 0.0 | 0.0 | 0.0 | 0.0 |
| Unilateral LG (n=14) | 0.0 | 0.214 | 0.0 | 0.786 | 0.0 | 0.0 | 0.0 | 0.0 |
Single-center retrospective observational study
Prenatal low-grade hydronephrosis without additional findings indicative for obstruction never led to an etiological diagnosis
203 interdisciplinary examinations
175 patients included and followed
Schürch et al. 2021
CONCLUSION: Prenatal involvement of pediatric nephrologists/urologists led to a high accuracy of diagnoses and may justify a restriction of follow-up to children with high-grade hydronephrosis or children with low-grade hydronephrosis plus additional findings indicative for obstruction.
